# The Volume Ratio of Ground Glass Opacity in Early Lung CT Predicts Mortality in Acute Paraquat Poisoning

**DOI:** 10.1371/journal.pone.0121691

**Published:** 2015-04-01

**Authors:** Xin Kang, Da-Yong Hu, Chang-Bin Li, Xin-Hua Li, Shu-Ling Fan, Yong Liu, Guang-Yu Tang, Zi-Sheng Ai, Tianfu Wu, Chandra Mohan, Xin J. Zhou, Jun-Yan Liu, Ai Peng

**Affiliations:** 1 Department of Nephrology & Rheumatology, Shanghai Tenth People's Hospital, Tongji University School of Medicine, Shanghai, PR China; 2 Department of Radiology, Shanghai Tenth People's Hospital, Tongji University School of Medicine, Shanghai, PR China; 3 Department of Medical Statistics, College of Medicine, Tongji University School of Medicine, Shanghai, PR China; 4 Department of Biomedical Engineering and Pharmacy, University of Houston, TX, United States of America; 5 Renal Path Diagnostics, Pathologists BioMedical Laboratories & Department of Pathology, Baylor University Medical Center, Dallas, TX, United States of America; Azienda Ospedaliero-Universitaria Careggi, ITALY

## Abstract

**Background:**

Pulmonary injury is the main cause of death in acute paraquat (PQ) poisoning. However, whether quantitative lung computed tomography (CT) can be useful in predicting the outcome of PQ poisoning remains unknown. We aimed to identify early findings of quantitative lung CT as predictors of outcome in acute PQ poisoning.

**Methods:**

Lung CT scanning (64-slide) and quantitative CT lesions were prospectively measured for patients after PQ intoxication within 5 days. The study outcome was mortality during 90 days follow-up. Survival curves were derived by the Kaplan-Meier method, and mortality risk factors were analyzed by the forward stepwise Cox regression analysis.

**Results:**

Of 97 patients, 41 (42.3%) died. Among the eight different types of lung CT findings which appeared in the first 5-day of PQ intoxication, four ones discriminated between survivors and non-survivors including ground glass opacity (GGO), consolidation, pneumomediastinum and “no obvious lesion”. With a cutoff value of 10.8%, sensitivity of 85.4% and specificity of 89.3%, GGO volume ratio is better than adopted outcome indicators in predicting mortality, such as estimated amount of PQ ingestion, plasma or urine PQ concentration, acute physiology and chronic health evaluation (APACHE) II and sequential organ failure assessment (SOFA) scores. GGO volume ratios above 10.8% were associated with increased mortality (hazard ratio, 5.82; 95% confidence interval, 4.77-7.09; *P* < 0.001).

**Conclusions:**

The volume ratio of GGO exceeding 10.8% is a novel, reliable and independent predictors of outcome in acute PQ poisoning.

## Introduction

Paraquat (PQ; 1,1-dimethyl-4,4-bipyridinium dichloride) is a highly toxic herbicide to humans [1–4]. Acute PQ poisoning during suicide attempts or after accidental ingestion causes a high mortality (50–90%) because of a lack of effective treatment or an antidote [3]. A uniformly used prognostic indicator that reliably predicts the risk of death at an early stage in poisoning is of great importance in assessing clinical benefit from various interventions [1, 2]. The main target organ for PQ toxicity is the lung as a consequence of PQ accumulation against a concentration gradient through the highly developed polyamine uptake system, and due to its capacity to generate huge amounts of pro-oxidant reactive species through a strenuous redox-cycle pathway [1, 2]. Most variables that are associated with mortality are non-specific to abnormalities of pulmonary pathophysiology, such as estimated amount of PQ ingestion [4], plasma and urine PQ concentration [5, 6], severity index for paraquat poisoning (SIPP) [7], acute physiology and chronic health evaluation II (APACHE II) and modified sequential organ failure assessment (mSOFA) scores [8, 9], liver enzymes, serum creatinine (SCr) and absolute lymphocyte count levels [10–12]. A few pulmonary indices, such as arterial blood bicarbonate and respiratory index, have been proposed as prognostic indicators [13, 14]; However, none has been validated independently or prospectively [2].

Chest computed tomography (CT) has been speculated to be useful in detecting early lung lesions and assessing long-term damage in PQ poisoned survivors [2]. In the 1990s, lm *et al*. [15] and Lee *et al*. [16] described the radiologic and high resolution CT (HRCT) manifestations of PQ-inducing pulmonary damage, with special emphasis on the sequential changes. In two separate retrospective studies, ground glass opacity (GGO) area and the number of lung segments involvement on CT have been claimed to correlate with the prognosis of PQ poisoning, respectively [17, 18]; However, the predictive relationship between early lung CT and mortality has not been established quantitatively yet.

Here, in this prospective nested case-control study, we presented evidence that GGO in early stage lung CT findings predicts the mortality of PQ intoxication qualitatively and quantitatively.

## Methods

### Study Design and Patients

This study was approved by Shanghai Tenth people's Hospital of Tongji University Institutional Review Board (IRB: 2010RES017), informed written consent was obtained from the patients or their nearest relatives.

One hundred and three consecutive patients admitted to our hospital through May 2010 to November 2013 with a history of acute PQ poisoning, as provided by patients or relatives, the transferring physician, or a pesticide bottle containing PQ, were enrolled in the study. All of the enrolled patients fulfilled the following inclusion criteria: (1) a history of oral PQ exposure; (2)15-75 years of age; (3) interval between PQ ingestion to admission < 24 h; and (4) plasma or urine PQ concentration exceeding 0.1 mg/L. Patients who had a history of serious lung disease, such as pulmonary tuberculosis and idiopathic pulmonary fibrosis, impaired liver or renal function, or a recent infectious disease, were excluded. Patients who died before baseline clinical information was collected or a CT measurement was performed were also excluded.

Standardized treatment protocols were conducted as follows. Briefly, after gastric lavage, emergency hemoperfusion 2–6 hours was performed if blood biochemistry tests showed impaired respiratory, renal or hepatic function, specifically, a serum creatinine level > 123.8 μmol/L, aspartate aminotransferase (AST) and alanine aminotransferase (ALT) value levels > 70 U/L, or a total bilirubin level > 36.6 μmol/L [19]. Methylprednisolone pulse treatment, glutathione and vitamin C antioxidant supplementation, prevention and treatment of infection, maintenance of water, electrolyte, and acid-base balance, protection of vital organs, and other symptomatic support therapies were initiated after admission. Hypoxia was diagnosed if a patient had an arterial blood gas PaO_2_ < 70 mmHg on room air [19]. Oxygen therapy was not provided, except as a palliative measure in patients in terminal decline.

Clinical outcome was defined as survivors or non-survivors. The survivors were described as patients who survived for 90 days after PQ ingestion and whose arterial blood gas analysis, serum creatinine level, and liver function test findings were all within the normal range after that time interval.

### Clinical Data Collection

Upon arrival at the emergency room, blood and urine samples were obtained from patients to determine PQ levels. Plasma and urine concentrations of PQ were determined by high performance liquid chromatography (HPLC), modified method previously reported by Whitehead et al [20]. We estimated the amount of PQ ingestion as follows: 5 ml = a small sip; 20 ml = a mouthful [21]; and the bottle liquid level change when the ingestion was large. In addition to routine examinations of blood and urine, the APACHE II and SOFA scores were also performed at the time of admission and were repeated every 2–3 days. We chose the maximum or minimum of these clinical parameters in the first 5-day of intoxication to analyze.

### CT Examinations

Lung CT examinations were performed using 64-slice CT angiography (Light Speed VCT; GE, USA) within 2 h of admission and at 2–3 days intervals during the first 5-day after PQ ingestion. Gases were excluded and the scan range was set from the apex to the base of the lung, allowing a full view of the lungs. Standard lung algorithm settings were used, as follows: mediastinal window (window width, 350 HU; window level, 40 HU); lung window (window width, 1350 HU; window level, -350 HU); and slice thickness (1.625 mm) and interlayer spacing (1.625 mm). Then, CT images were reconstructed at a slice thickness of 5 mm. Two sequences of lung and mediastinal windows were obtained during examination of each patient. No contrast agent was injected for enhancement scanning.

**Fig 1:**
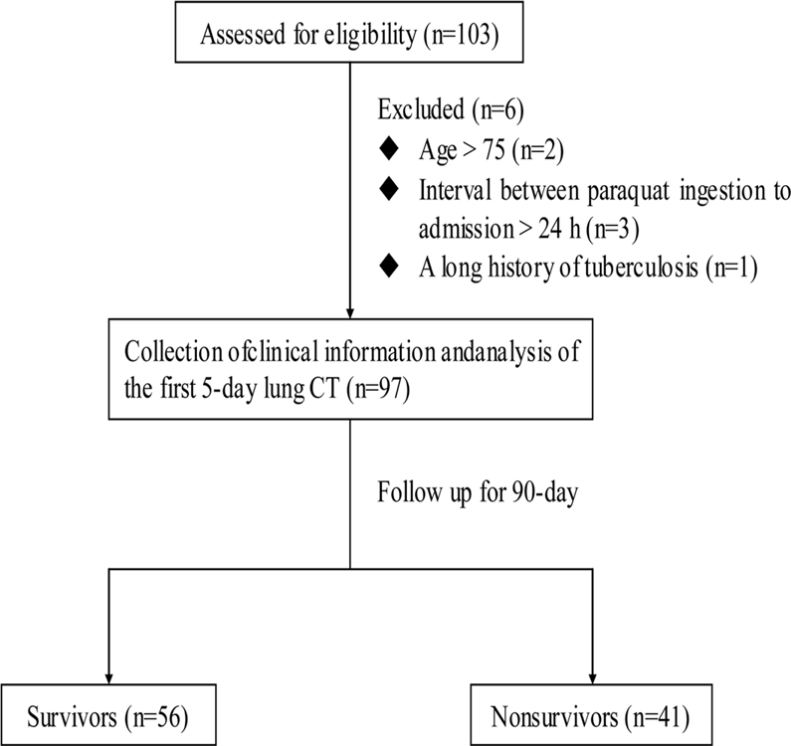
Flow chart.

Observers were board-certified thoracic radiologists with 20 and 18 years of experience, respectively, and were blind to clinical data and outcome of patients. The CT images were assessed independently by using CT work-station ADW4.6 (GE, USA). The adopted CT diagnoses for further analyses were those agreed upon by consensus between the two radiologists. The CT findings during the first 5-day of intoxication were divided into eight types, as follows: GGO; consolidation; pleural thickening; hydrothorax; fibrosis; pneumomediastinum or subcutaneous pneumomediastinum; nodules [22], and “no obvious lesion” which was defined as all CT performed during the first 5-day were diagnosed “nothing abnormal detected”. (Fig. 1)

Once the lung is segmented, the computer is used to count the number of voxels within the lung region. Then the lung volume can be obtained by multiplying the number of voxels by the voxel dimensions [23]. The voxel dimensions in the X and Y planes are the field of view (FOV) divided by the image matrix size (512 × 512). The voxel dimension in the Z dimension is the slice thickness. Similarly, the observers manually traced the contour of the lesions on each CT slice and all lesion volumes were calculated [24]. Then the observers used the lesion volume ratio adjusted by the whole lung volume. As for the pneumomediastinum or subcutaneous emphysema, we did not perform the quantification because its volume hard to adjust. The quantitative CT measurements were also conducted independently by the observers. In this study, the mean value of lesion volume ratios from the two observers were represented as the magnitude of the lesions and the maximum of the lesion volume ratios observed during the first 5-day following PQ intoxication was chosen for further analysis.

### Statistical Analysis

Data are presented as absolute numbers, percentages, mean (±SD), or median (interquartile range). Comparisons of continuous variables were made using the Mann-Whitney U test. Categorical data were compared using a chi-square test. Receiver operating characteristic (ROC) curves were computed and areas under the curves were used to evaluate how well a model distinguishes the survivor group from the non-survivor group. A multivariate Cox proportional hazards regression model with forward stepwise selection procedures was used to identify risk factors for 90-day mortality. A *P* value < 0.1 in the univariate analysis was required for a variable to enter the multivariate model. The survival analysis was performed using the Kaplan-Meier method and log-rank test. All analyses were performed using SPSS 20.0 for Microsoft Windows (SPSS, Inc., Chicago, IL, USA). A *P* value < 0.05 was considered statistically significant.

## Results

### Characteristics of the PQ poisoned Patients

**Fig 2:**
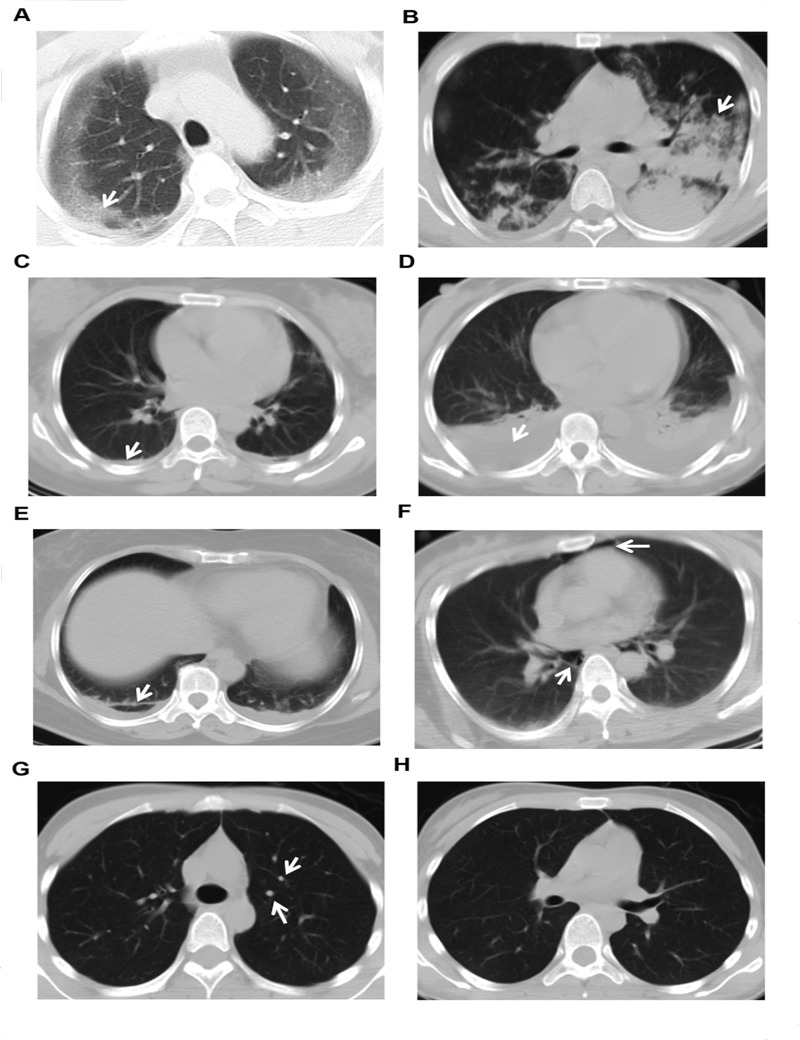
Eight CT findings of PQ poisoning captured within the first 5-day. A. ground glass opacity (GGO); B. consolidation; C. pleural thickening; D. hydrothorax; E. fibrosis; F. pneumomediastinum; G. nodule; H. “no obvious lesion”. Arrows represented the lesions.

Among the 103 patients, 97 were enrolled for analysis; 5 patients did not meet inclusion criteria, another one were excluded because of a long history of pulmonary tuberculosis, and none was lost to follow-up. (Fig. 2)

Defined by status on day 90 after PQ ingestion, there were 56 (57.7%) survivors and 41 (42.3%) non-survivors. Comparing survivors with non-survivors, we found the following: (1) age, gender, and arrival time at the hospital after ingestion were not significantly different; (2) the estimated amount of PQ ingested, the PQ concentration in the plasma and urine, and the clinical scores of the non-survivors were higher significantly than those of survivors (*P* < 0.001); (3) the difference in frequency of nausea and vomiting (*P* = 0.002) and chest congestion and tachypnea (*P* < 0.001) were more significant in non-survivors than in survivors, compared to other symptoms such as oral burning sensation, sore throat and stomach ache; (4) non-survivors had a higher incidence of ARF, toxic hepatitis, and hypoxemia than survivors (*P* < 0.001); (5) the BUN, SCr, AST, ALT, total bilirubin, and direct bilirubin levels were significantly higher among non-survivors while the pH (*P* = 0.002) and PaO_2_ (*P* < 0.001) of the non-survivor group were significantly lower. In contrast the PaCO_2_ was not statistically significant; and (6) the percentage of non-survivors who underwent hemoperfusion was higher than survivors (*P <* 0.001) (Table 1).

**Table 1:** Demographic and Clinical Data of Patients during the first 5-day of PQ Poisoning.

	Survivors *(n = 56)*	Nonsurvivors *(n = 41)*	*P* value
**Age, *year***	34.3±10.8	33.5±12.5	0.289
**Male/Female, n**	18/38	18/23	0.121
**Time to hospital after ingestion, *hour***	14.3±5.5	14.1±5.9	0.880
**Estimated PQ amount, *ml***	15 (10–20)	50 (20–110)	< 0.001
**Plasma PQ concentration, *mg/L***	0.1 (0.0–0.2)	0.8 (0.1–6.7)	< 0.001
**Urine PQ concentration, *mg/L***	1.5 (0.1–5.6)	23.7 (8.9–267.0)	< 0.001
**Symptom**			
** Burning sensation, n (%)**	36 (64.3)	23 (56.1)	0.414
** Sore throat, n (%)**	31 (55.4)	29 (70.7)	0.124
** Nausea and vomiting, n (%)**	13 (23.2)	22 (53.7)	0.002
** Stomachache, n (%)**	9 (16.1)	13 (31.7)	0.069
** Chest stuffy and tachypnea, n (%)**	0 (0.0)	17 (41.5)	< 0.001
**Scoring**			
** APACHE II**	3 (1–5)	10 (5–12)	< 0.001
** SOFA**	2 (0–4)	5 (3–7)	< 0.001
**Renal function**			
** ARF, n (%)**	15 (26.8)	37 (90.2)	< 0.001
** Serum blood urea nitrogen, *mmol/L***	5.1 (3.1–9.5)	7.9 (6.0–13.0)	< 0.001
** Serum creatinine, *umol/L***	68.5 (49.3–138.8)	222.0 (157.6–301.0)	< 0.001
**Liver function**			
** Toxic hepatitis, n (%)**	4 (7.1)	29 (70.7)	< 0.001
** Serum AST, *IU/L***	21.5 (16.2–39.8)	185.7 (71.2–451.8)	< 0.001
** Serum ALT, *IU/L***	17.0 (10.0–44.7)	217.9 (38.5–346.4)	< 0.001
** Serum total bilirubin,** μ***mol/L***	14.9 (8.4–19.5)	35.7 (10.2–66.7)	< 0.001
** Serum direct Bilirubin,** μ***mol/L***	6.3 (3.5–8.4)	23.1 (5.4–48.6)	< 0.001
**Arterial blood gases**			
** Hypoxemia, n (%)**	2 (3.6)	28 (68.3)	< 0.001
** PH**	7.40 (7.37–7.42)	7.31 (7.26–7.41)	0.002
** PaO**_**2**_ **, *mmHg***	95.5 (87.9–102.0)	55.7 (51.5–73.6)	< 0.001
** PaCO**_**2**_ **, *mmHg***	37.2 (33.8–40.0)	33.0 (27.9–40.4)	0.169
**Hemoperfusion, n (%)**	23 (41.1)	38 (92.7)	< 0.001

*Definition of abbreviations*: PQ = paraquat; APACHEII = Acute Physiology and Chronic Health Evaluation; SOFA = Sequential Organ Failure Assessment; continuous variable are presented as means ± SD or median (interquartile range) and categorical variable is presented as no. (%).

Note: the values of Symptom, Scoring, renal function, liver function and arterial blood gases were the peak values during the first 5-day.

### Early Lung CT Findings

**Fig 3:**
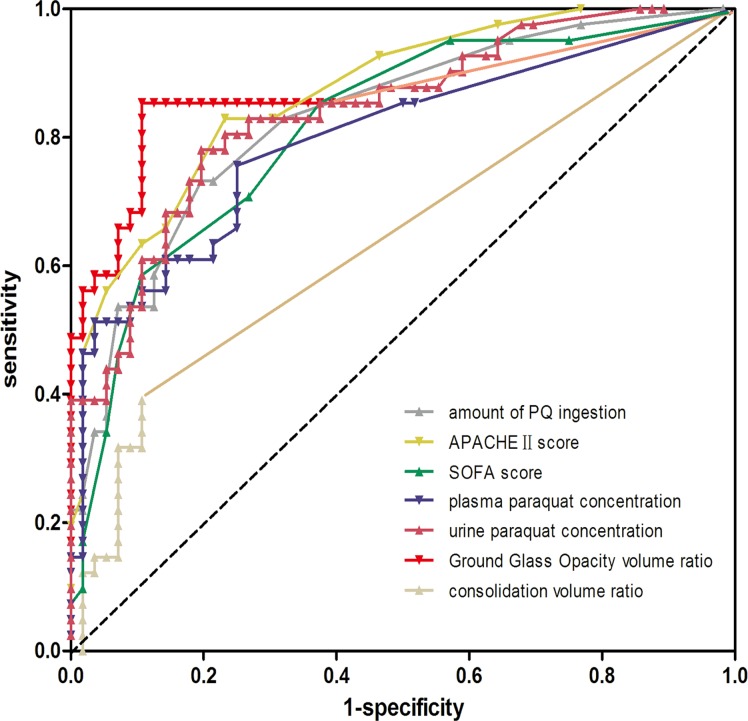
Kaplan-Meier survival curves of 97 paraquat poisoned cases stratified according to different CT findings within the first 5-day period. Patients were categorized into two groups based on whether or not they presented the specific CT finding. The P values were derived by log-rank test.

Among the eight lung CT findings within 5 days after PQ ingestion, GGO (odds ratio [OR] = 9.015, *P* < 0.001), consolidation (OR = 5.333, *P* = 0.001), and pneumomediastinum (OR = 15.469, *P* < 0.001) exhibited significantly higher incidences in the non-survivor group than the survivor group. In contrast, CT scans with no obvious lesion were more frequent in the survivor group (OR = 0.154, *P* = 0.002). Other lesions, such as pleural thickening, hydrothorax, fibrosis, and nodules, were slightly different between the two groups (Table 2). The timing of death events for the eight CT findings is shown in Fig. 3. The patients with three lung lesions including GGO, consolidation and pneumomediastinum, significantly shortened the death time (P < 0.05).

**Table 2:** Eight Lung CT Findings during the First 5 Days after PQ Intoxication.

CT finding	Survivors (*n = 56*)	Nonsurvivors (*n = 41*)	*P* value	*OR*
**Ground glass opacity, (%)**	22 (39.3)	35 (85.4)	< 0.001	9.015
**Consolidation, (%)**	6 (10.7)	16 (39.0)	0.001	5.333
**Pleural thickening, %**	17 (30.4)	8 (19.5)	0.228	0.566
**Hydrothorax, (%)**	12 (21.4)	7 (17.1)	0.593	0.755
**Fibrosis, (%)**	21 (37.5)	8 (19.5)	0.056	0.404
**Pneumomediastinum, (%)**	1 (1.8)	9 (22.0)	< 0.001	15.469
**Nodule, (%)**	10 (17.9)	4 (9.8)	0.262	0.497
**“No obvious lesion”, (%)**	19 (33.9)	3 (7.3)	0.002	0.154

*Definition of abbreviations*: PQ = paraquat; OR = odds ratio.

### ROC Curve Analysis for Mortality

**Fig 4:**
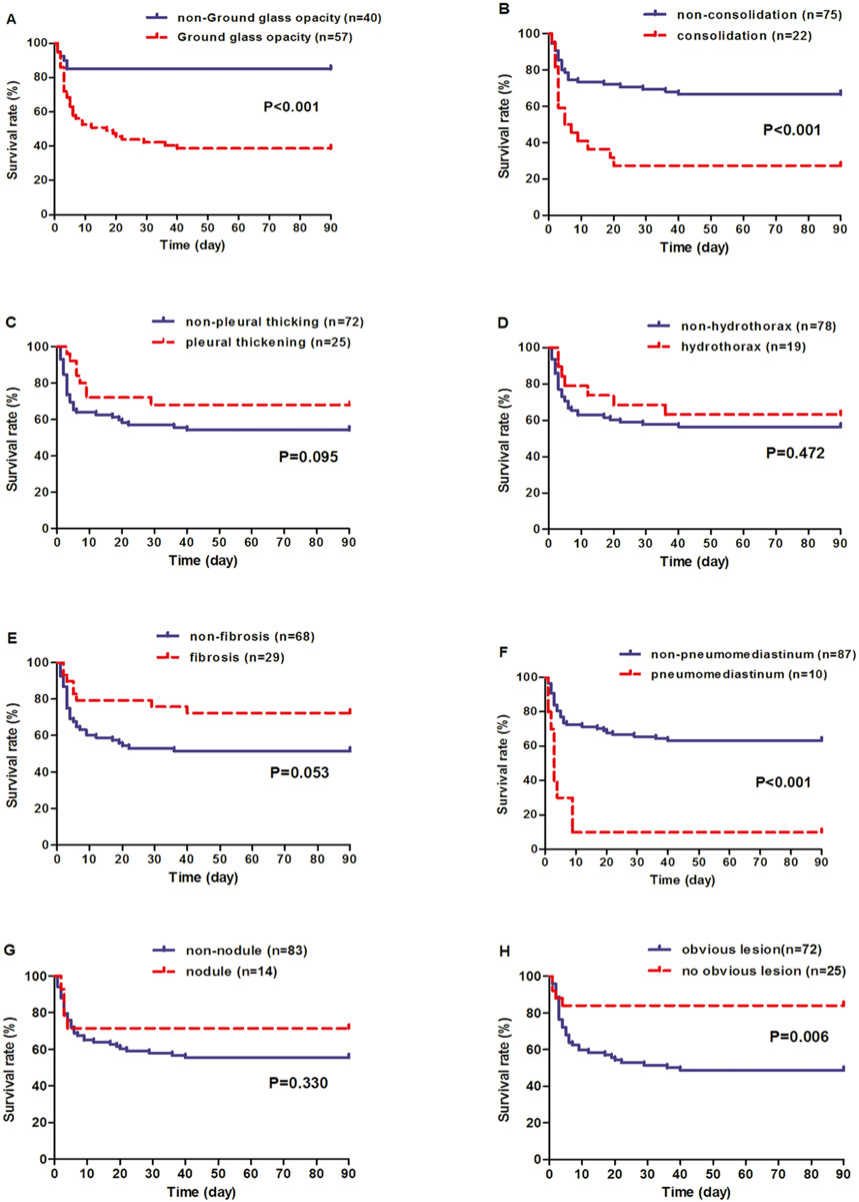
The receiver operating characteristic (ROC) curves constructed for outcome prediction by amount of paraquat (PQ) ingestion, plasma and urine concentrations of PQ, scores of Acute Physiology and Chronic Health Evaluation (APACHE) II and Sequential Organ Failure Assessment (SOFA), as well as volume ratio of Ground glass opacity and consolidation following PQ poisoning.

The prediction of PQ poisoning associated mortality based on the estimated amount of PQ ingested, and PQ concentration, as well as scores and volume ratios of GGO and consolidation was determined based on ROC curves (Fig. 4).

The area under the ROC curve for the GGO volume ratio (areas under curve [AUC] = 0.871, *P* < 0.001) was larger than that for APACHEII(AUC = 0.856, *P* < 0.001), SOFA scores (AUC = 0.788, *P* < 0.001), estimated PQ ingestion (AUC = 0.850, *P* < 0.001) and plasma PQ levels (AUC = 0.826, P < 0.001), and urine PQ concentrations (AUC = 0.861, P < 0.001). However, no significant difference was observed for the volume ratio of consolidation (AUC = 0.621, *P* = 0.096). We also used the ROC curves to determine the optimal cut-off points of these parameters for predicting the 90-day mortality; the prediction sensitivity and specificity, positive predictive value, negative predictive value, and accuracy for each parameter and individual threshold are presented in Table 3.

**Table 3:** Comparison of Predictors for Mortality of PQ Poisoning.

Variable	Cutoff	AUC (95% CI)	Sen	Spe	PPV	NPV	ACC
**Amount of PQ ingestion**	27.5ml	0.850 (0.837–0.863)	0.732	0.804	0.732	0.804	0.773
**APACHE II Score**	4.5	0.856 (0.843–8.868)	0.829	0.768	0.723	0.860	0.794
**SOFA Score**	2.5	0.788 (0.773–0.807)	0.854	0.732	0.700	0.872	0.784
**Plasma PQ concentration**	0.8mg/L	0.826 (0.812–0.841)	0.512	0.964	0.909	0.720	0.763
**Urine PQ concentration**	8.3mg/L	0.861 (0.849–0.874)	0.780	0.804	0.727	0.830	0.784
**Volume ratio of GGO**	10.8%	0.871 (0.857–0.884)	0.854	0.893	0.812	0.893	0.876
**Volume ratio of consolidation**	7.1%	0.634 (0.614–0.653)	0.390	0.829	0.765	0.650	0.670

*Definition of abbreviations*: PQ = paraquat; AUC = area under a receiver operator curve; Sen = sensitivity; Spe = specificity; PPV = positive predictive value; NPV = negative predictive value; ACC = Accuracy; APACHEII = Acute Physiology and Chronic Health Evaluation; SOFA = Sequential Organ Failure Assessment;

Note: amount of PQ ingestion, plasma PQ concentration, and urine PQ concentration were collected on admission; other parameters were the peak values within 5 days following intake of paraquat.

### Early Lung CT Findings as Independent Predictors for Mortality

Except for estimated amount of PQ ingestion, plasma and urine concentrations of PQ, scores of APACHEII and SOFA, the four CT findings including GGO, consolidation, pneumomediastinum and “no obvious lesion” were also associated with outcome for PQ poisoning, and a univariate Cox regression analysis supported the prognostic value of these variables. GGO (hazard ratio [HR] = 5.82; 95% confidence interval [CI], 4.77–7.09; *P* < 0.001) consolidation (P = 0.229), and pneumomediastinum (HR = 1.72; 95% CI; 1.49–1.98; *P* < 0.001) were still associated with an increased risk of death after adjusting for the estimated amount of PQ ingestion, plasma and urine concentrations of PQ, scores of APACHEII and SOFA. (Table 4).

**Table 4:** Cox Proportional Hazards Models for Mortality Prediction of PQ Poisoning.

	Univariate Cox Model		Multivariate Cox Model	
**variable**	HR (95%CI)	*P* value	HR (95%CI)	*P* value
**Amount**	6.91 (6.11–7.81)	< 0.001	2.49 (2.15–2.88)	< 0.001
**Plasma**	10.21 (9.17–11.49)	< 0.001	4.52 (3.92–5.21)	< 0.001
**Urine**	7.81 (6.86–8.89)	< 0.001	2.04 (1.74–2.40)	< 0.001
**APACHEII**	8.08 (6.99–9.33)	< 0.001	3.80 (3.24–4.46)	< 0.001
**SOFA**	5.23 (4.54–6.02)	< 0.001	N/A	0.225
**Ground glass opacity**	14.34 (12.23–16.80)	< 0.001	5.82 (4.77–7.09)	< 0.001
**Consolidation**	2.74 (2.45–3.08)	< 0.001	N/A	0.229
**Pneumomediastinum** *	6.09 (5.31–6.99)	< 0.001	1.72 (1.49–1.98)	< 0.001
**“No obvious lesion”** *	0.35 (0.30–0.42)	< 0.001	N/A	0.240

*Definition of abbreviations*: PQ = paraquat; HR = harzard ratio; N/A = not applicable.

Note: * indicates variables were categorical using the presence of CT finding as a dichotomous variable; other variables were categorical according whether lesion volume ratio was above or below the optimal cutoff point.

**Fig 5:**
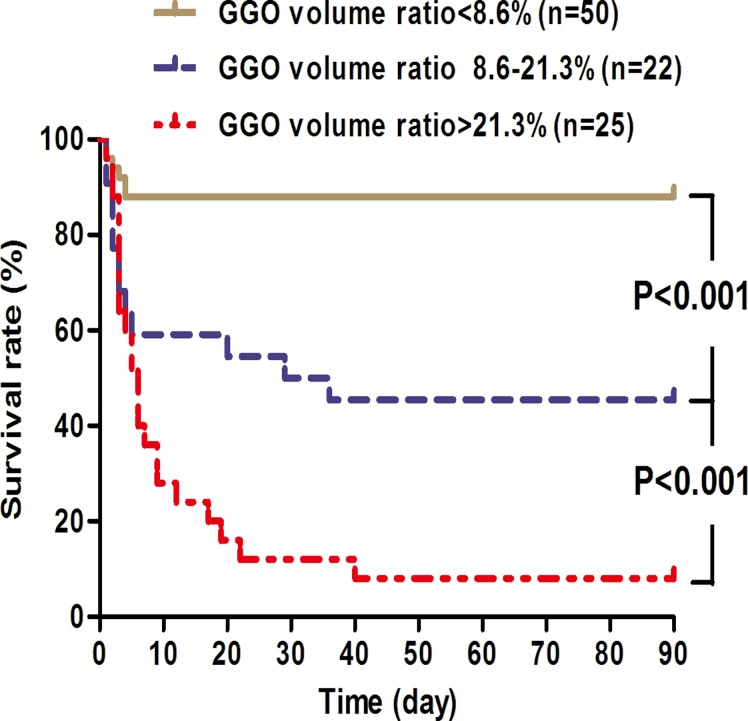
Kaplan-Meier survival curves of 97 paraquat poisoned cases stratified according to levels of GGO volume ratios within the first 5-day period. Patients were categorized into three groups based on quartile levels of GGO volume ratios. Comparisons between curves showing difference with statistical significance (P < 0.05) are indicated.

The additional prognostic value of the GGO volume ratio is further illustrated in the Kaplan-Meier survival curve (Fig. 5), patients were categorized according to quartiles of GGO volume ratio levels to reduce the right skewness, and high levels of the GGO volume ratio indicated a patient population with dismal prognosis (*P* < 0.001).

## Discussion

This prospective study showed that four lung CT findings (GGO, consolidation, pneumomediastinum, and “no obvious lesion”) were significantly different between survivors and non-survivors within the first 5-day of PQ ingestion (Table 2). In addition, patients with first three CT findings had significantly higher mortality based on Kaplan-Meier survival curves, while patients with “no obvious lesion” indicated a higher survival rate (Fig. 3). More importantly, GGO and pneumomediastinum independently predicted the 90-day mortality based on multivariate Cox regression (Table 4), and a volume ratio of GGO higher than 10.8% was associated with a higher probability of mortality in patients with PQ poisoning (Fig. 5). Together, these results strongly suggest that early lung CT findings are associated with the death of PQ intoxication and the quantitative GGO volume ratio serves as an independent indicator for outcome.

Interestingly, the discriminatory power of the quantitative GGO volume ratio was better in predicting mortality following PQ poisoning than the estimated amount of PQ ingestion, PQ concentration, APACHEIIand SOFA scores by ROC curve analysis in the current study (Fig. 4). Using the GGO volume ratio (cut-off value = 10.8%) within the first 5-day of intoxication, the clinical sensitivity was 85.4%, the specificity was 89.3%, and the diagnostic accuracy was 87.6% with a negative predictive value of 89.3% and a positive predictive value of 81.2% (Table 3). These data suggest that the early quantitative GGO lesion is a novel, reliable and independent predictor in PQ poisoning.

Kim *et al* enrolled 119 PQ poisoned patients and reported that the GGO area on chest CT in the 7^th^ day after intoxication is a useful predictor of survival in acute PQ intoxication [17]. However, they excluded 131 patients, more than enrolled ones, who died or was discharged hopelessly within 7 days after intoxication, The GGO area for these patients remained unknown. In addition, the calculation of GGO area for the whole lung was measured at only five marked levels and only one time in the 7^th^ day and the other CT lesions was not analyzed. Thus the considerable amount of CT information was overlooked and the prediction of GGO area was not compared with other indicators such as PQ concentration *etc*. By contrast, this prospective study, the CT information was collected during the first 5 days in PQ intoxication. More importantly, the quantitative volume ratios of CT lesions were adjusted by the whole pulmonary volume. Thus this study was the first one to quantitatively establish the prediction of 5-day lung CT findings for PQ poisoning, and quantify the lung lesions more accurately.

We did not choose the time interval earlier or analyze CT performance separately during the first 5-day, because in the first 96h, 41.2% of patients (26 in survivors *vs*. 14 in nonsurvivors) showed “no obvious lesion” (seen in S1 Table) and through the 96h to 5-day, the incidence of GGO reached up to 90.5% in the non-survivor because 20 patients had died in the first 96h (seen in S2 Table). So if we chose the first 96h through which the nearly 1/2 patients showed no obvious CT lesion, the prediction of CT for outcome was inaccurate. If we focused the 96h to 5-day, the reduced nonsurvivors resulted from the early death in the first 96h probably exaggerate some CT findings’ prediction such as GGO or cover up the importance of some CT findings such as PM. Therefore, we eventually analyzed CT performance during the first 5-day.

It is well-known that the main target organ for PQ toxicity is the lung [1, 2]. The pulmonary lesion has two phases, acute alveolitis followed by secondary fibrosis. In the alveolitis phase, the air-blood barrier is destroyed; type I and II pneumocytes swelling, thus the gas exchange between the air space and the capillaries is destroyed and the surface tension within the alveoli is increased, causing edema of capillaries. Damaged pulmonary capillary endothelium leads to pulmonary hemorrhage or edema and infiltration in the interstitial tissue and air spaces of the lung with inflammatory cells. The onset of the proliferative phase occurs several days after PQ ingestion. The normal architecture of the lung is destroyed due to the proliferation of fibroblasts and deposition of collagen, thereby reducing the effectiveness of gaseous exchange, leading to death as a consequence of severe anoxia [1, 2]. In a previous study, the GGO lesion appears during the damage stage and reflects alveolar edema and inflammatory cell infiltration, thus indicating primary alveolitis from PQ [1, 15]. Therefore, the quantitative GGO lesion may represent the extent of alveolitis and could predict the risk of death in the early stage. Together, these pathologic findings demonstrated that GGO could be a pulmonary-specific predictor for mortality following PQ poisoning. Although pulmonary fibrosis is known to cause progressive respiratory failure and death [4, 25], in the present study lung fibrosis in CT was not significantly associated with the outcome of PQ poisoning, which may be explained by the fact that fibrosis peaks in the proliferative phase and all the data of CT in our study was collected within the first 5 days after PQ intoxication.

In our study, ten patients presented with pneumomediastinum, and 9 of them succumbed within 5 days of appearance of this lesion. Pneumomediastinum was also shown to be an independent outcome indicator based on multiple Cox regression. To date, there were a total of 23 cases of pneumomediastinum in PQ poisoning reported [15, 26–30], 17 of these patients were reported in 1991 by Im *et al* [14], and the other 6 cases was reported in case reports [26–30]. Out of 23 patients, 20 died within 3 days of its appearance [15, 26, 27]. These previous studies and our own findings all suggest pneumomediastinum could predict bad outcome and short survival time. Hence, we believe that the prognostic value of this CT lesion in this setting deserves further investigation.

The quantitative GGO lesion on early-stage lung CT may well complement or improve upon other widely adopted indicators in outcome prediction, for the following reasons: (1) The amount of PQ ingestion may be a direct method to predict outcome, but an accurate amount of ingestion cannot always be provided by patients and their relatives [1, 2]; (2) PQ concentration is most commonly used to predict outcomes in PQ poisoning and demonstrates excellent discrimination power in evaluating prognosis over a short time (generally 6~24 h) after PQ ingestion [5]; However, the majority of patients are beyond the window of detection and prediction is not good when the plasma PQ level is low [31]. (3) Alternative clinical parameters like APACHEIIand SOFA scores have good significance according to recent studies [8, 9], but the scoring is frequently variable and can be affected by multiple organ function, for example Glasgow Coma Scale (GCS) is an important estimation project in both scores although PQ poisoned patients are always sanity [8, 9]; (4) Lung CT measurement is easily accessible and can be accomplished with high reproducibility, and the time constraint for prediction is less stringent than detection of PQ concentration. (5) More importantly, outcome can be predicted accurately and easily by the GGO volume ratio using quantitative CT measures, and GGO lesions on lung CT are pulmonary-specific.

To rule out confounding factors, our study was prospective design, with a standard protocol, broad spectrum of clinical characteristics of the patients and quantitative measurement of PQ concentration by HPLC for all patients. Moreover, we used quantitative lung CT technology for accurate and reliable outcome prediction. Quantitative lung measurement is used commonly [32] and there is a uniform agreement that lung volume can be reliably and accurately estimated using CT scans [33]. We quantified the CT volume and specific lesion volume manually, which is considered more accurate than automatic methods [22]. The results of quantitative CT measurements were corroborated by two experienced radiologists, thereby reducing the observer subjectiveness. We did not conduct testing of biomarkers for acute lung injury; However, we consider it unlikely that this could have affected the results because no pulmonary-specific biomarker has been reported to be associated with PQ poisoning. Finally the current study was monocentric, further multicentric investigation is warranted.

## Conclusions

In summary, we found quantitative GGO volume ratio in early stage emerges as a novel and promising predictor for mortality in acute PQ poisoning, which may provide clinicians with useful early prognostic information that could guide treatment strategies.

## Supporting Information

S1 Table Eight Lung CT Findings during the first 96 hours after Paraquat (PQ) Intoxication. (DOCX)

S2 Table Eight Lung CT Findings during the 96 hours—5 days after Paraquat (PQ) Intoxication. (DOCX)
