# Supplementary material for: The Volume Ratio of Ground Glass Opacity in Early Lung CT Predicts Mortality in Acute Paraquat Poisoning
Source: PLoS One. 2015 Apr 1;10(4):e0121691. doi: 10.1371/journal.pone.0121691 (PMC4382148; doi:10.1371/journal.pone.0121691)
Supplement: S1 Table — (DOCX) [file pone.0121691.s001.docx]

**S1 Table. Eight Lung CT Findings during the first 96 hours after paraquat (PQ) Intoxication.**

| **CT finding** | **Survivors (*n=56*)** | **Nonsurvivors (*n=41*)** | ***P* value** | ***OR*** |
| --- | --- | --- | --- | --- |
| **Ground glass opacity (GGO) , %** | 13 (23.2) | 27 (65.9) | <0.001 | 6.379 |
| **Consolidation , %** | 4 (7.1) | 13 (31.7) | 0.002 | 6.036 |
| **Pleural thickening , %** | 15 (26.8) | 6 (14.6) | 0.151 | 1.147 |
| **Hydrothorax , %** | 9 (16.1) | 5 (12.2) | 0.592 | 0.752 |
| **Fibrosis , %** | 17 (30.3) | 6 (14.6) | 0.072 | 0.393 |
| **Pneumomediastinum , %** | 0 (0.0) | 8 (19.5) | 0.000 | NA |
| **Nodule , %** | 8 (14.3) | 4 (9.8) | 0.503 | 0.649 |
| **“No obvious lesion” , %** | 26 (46.4) | 14 (34.1) | 0.225 | 0.598 |

*Definition of abbreviations*: PQ = paraquat; OR = odds ratio.
