# Supplementary material for: The Volume Ratio of Ground Glass Opacity in Early Lung CT Predicts Mortality in Acute Paraquat Poisoning
Source: PLoS One. 2015 Apr 1;10(4):e0121691. doi: 10.1371/journal.pone.0121691 (PMC4382148; doi:10.1371/journal.pone.0121691)
Supplement: S2 Table — (DOCX) [file pone.0121691.s002.docx]

**S2 Table. Eight Lung CT Findings during the 96 hours - 5 days after paraquat (PQ) Intoxication.**

| **CT finding** | **Survivors (*n=56*)** | **Nonsurvivors (*n=21*)** | ***P* value** | ***OR*** |
| --- | --- | --- | --- | --- |
| Ground glass opacity (GGO) , % | 22 (39.3) | 19 (90.5) | < 0.001 | 14.682 |
| Consolidation , % | 6 (10.7) | 7 (33.3) | 0.044 | 4.167 |
| Pleural thickening, % | 17 (30.4) | 7 (33.3) | 0.802 | 1.147 |
| Hydrothorax , % | 12 (21.4) | 5 (23.8) | 1.000 | 1.146 |
| Fibrosis, % | 21 (37.5) | 5 (23.8) | 0.256 | 0.521 |
| Pneumomediastinum , % | 1 (1.8) | 2 (9.5) | 0.121 | 5.789 |
| Nodule, % | 10 (17.9) | 1 (4.8) | 0.273 | 0.230 |
| “No obvious lesion” , % | 19 (33.9) | 1 (4.8) | 0.009 | 0.097 |

*Definition of abbreviations*: PQ = paraquat; OR = odds ratio.
